# Crystal structure of the major quadruplex formed in the promoter region of the human c-MYC oncogene

**DOI:** 10.1371/journal.pone.0205584

**Published:** 2018-10-12

**Authors:** Sascha Stump, Tung-Chung Mou, Stephen R. Sprang, Nicholas R. Natale, Howard D. Beall

**Affiliations:** 1 Center for Environmental Health Sciences, Department of Biomedical and Pharmaceutical Sciences, University of Montana, Missoula, Montana, United States of America; 2 Center for Biomolecular Structure and Dynamics, Division of Biological Sciences, University of Montana, Missoula, Montana, United States of America; University of Iowa, UNITED STATES

## Abstract

The c-MYC oncogene mediates multiple tumor cell survival pathways and is dysregulated or overexpressed in the majority of human cancers. The NHE III_1_ region of the c-MYC promoter forms a DNA quadruplex. Stabilization of this structure with small molecules has been shown to reduce expression of c-MYC, and targeting the c-MYC quadruplex has become an emerging strategy for development of antitumor compounds. Previous solution NMR studies of the c-MYC quadruplex have assigned the major conformer and topology of this important target, however, regions outside the G-quartet core were not as well-defined. Here, we report a high-resolution crystal structure (2.35 Å) of the major quadruplex formed in the NHE III_1_ region of the c-MYC promoter. The crystal structure is in general agreement with the solution NMR structure, however, key differences are observed in the position of nucleotides outside the G-quartet core. The crystal structure provides an alternative model that, along with comparisons to other reported quadruplex crystal structures, will be important to the rational design of selective compounds. This work will aid in development of ligands to target the c-MYC promoter quadruplex with the goal of creating novel anticancer therapies.

## Introduction

Guanine-rich sequences of DNA and RNA can form a secondary nucleic acid structure known as a quadruplex. Quadruplex motifs have become the subject of significant interest due to their presence in human telomeres, 5’-untranstranslated regions of mRNA, and in gene promoter regions [1]. One such quadruplex-forming sequence is found in the promoter region of the human c-MYC oncogene. c-MYC is estimated to be dysregulated or overexpressed in approximately 70% of all human cancers and is responsible for mediating multiple pathways important in tumor cell survival [2]. Stabilization of the major quadruplex formed in the c-MYC promoter by various small molecules has been shown to inhibit transcription of c-MYC thereby reducing expression of the oncogene [3,4]. This reduction in c-MYC expression has been demonstrated to induce apoptosis in multiple types of tumor cells [5,6]. Taken together, these findings suggest that the c-MYC promoter quadruplex is a promising antitumor target. Several research groups are designing small molecules to stabilize the c-MYC promoter quadruplex as a strategy to develop potential therapies for treatment of human cancers [7–9].

**Fig 1:**
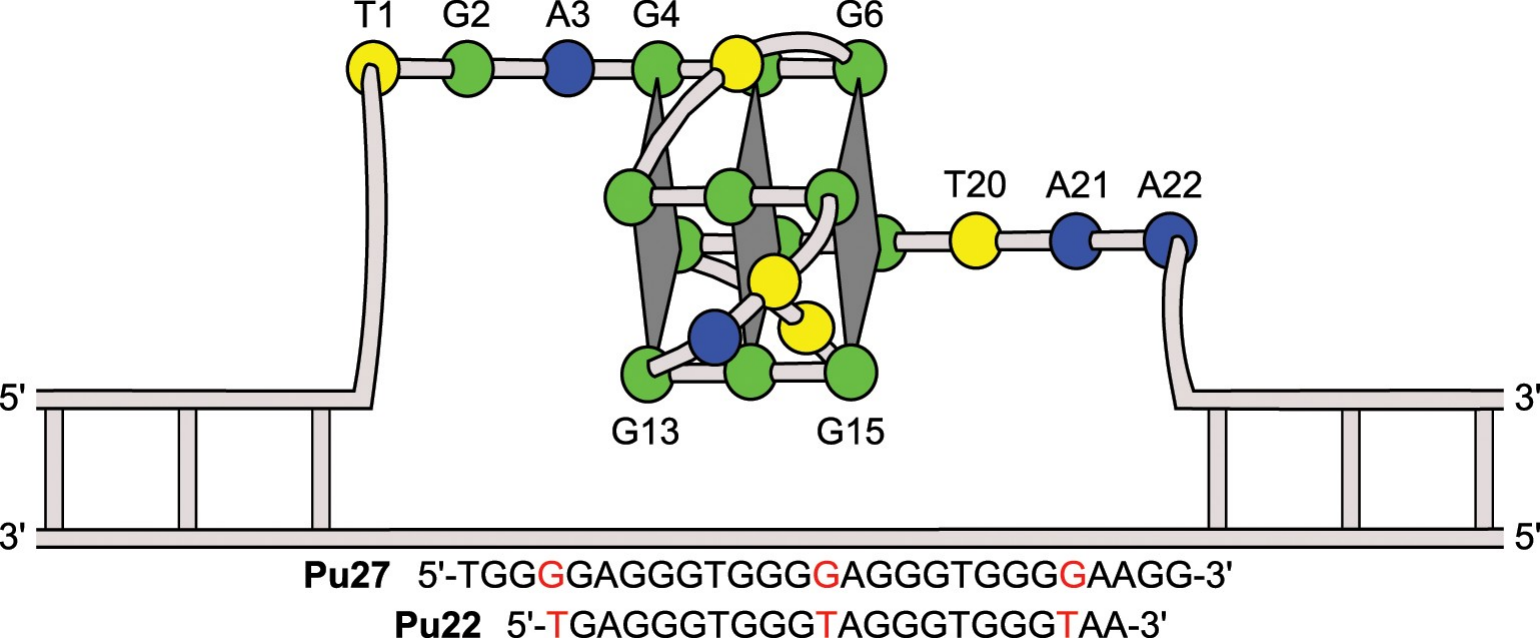
Topology of the c-MYC promoter quadruplex. Diagram showing fully parallel topology of the c-MYC promoter quadruplex crystal structure with all reversal loops continuing in the right to left direction (5’ to 3’). Thymines are represented in yellow, adenines in blue, and guanines in green. Sequence modifications in Pu22 are shown highlighted in red.

The general sequence motif that forms a quadruplex consists of several short guanine repeats (G), separated by short “loop” regions (L) comprised of other nucleotides with the overall general sequence of G_3-5_L_<7_G_3-5_L_<7_G_3-5_L_<7_G_3-5_ [1]. Quadruplexes can form as intramolecular or intermolecular arrangements, consisting of single or multiple nucleic acid strands, respectively. The basic unit of the quadruplex is the G-quartet, which is formed as a planar arrangement of four guanine residues held together through Hoogsteen bonding and stabilized by a central monovalent cation. Multiple G-quartets, usually three or more, stack upon each other to form the quadruplex secondary structure and are connected through external loop region nucleotides. The central channel cations are essential for quadruplex formation, with potassium generally preferred to sodium. Potassium cations are observed in a symmetric square antiprismatic coordination at the interface of two G-quartets, coordinated by the guanine O6 atoms, whereas the relatively smaller sodium atoms display square-planar coordination and are central to a single G-quartet. The species and abundance of these and other cations in solution can also serve to influence and stabilize the overall quadruplex topology [10]. Quadruplexes can be further categorized as parallel, anti-parallel, or hybrid by the types of loops formed and direction of the backbone in relation to the G-quartets (Fig 1) [1].

Parallel-type quadruplexes are found in promoter regions of c-MYC and several other oncogenes that are potential therapeutic targets for cancer including c-KIT, Bcl-2, VEGF, and HIF-1α [3,11–16]. The human c-MYC promoter quadruplex is formed under negative supercoiling conditions in the nuclease hypersensitivity element III_1_ region (NHE III_1_) [17]. NHE III_1_ is 27 residues in length and responsible for the regulation of 80–90% of c-MYC oncogene transcription (Pu27) [18]. Duplex/quadruplex or single-stranded forms of the NHE III_1_ region can be bound by transcription factors SP1 or CNBP/hnRNP, respectively, to increase c-MYC expression while formation of the quadruplex structure prevents transcription [19,20]. NHE III_1_ contains 20 guanines grouped into five segments of 3 to 4 guanine nucleotides separated by one or two adenine or thymine nucleotides. This guanine-rich character allows formation of four possible quadruplex topologies, with the major form being a parallel intramolecular quadruplex comprised of the four guanine segments at the 3’-end of the sequence [18].

In a previous NMR study, it was found that the major biologically relevant conformation adopted by the c-MYC promoter quadruplex could be selected from other conformers by mutating residues G4, G14 and G23 to thymines and truncating the sequence to 22 nucleotides in length (Pu22) [18]. In a separate NMR study, the binding of a quindoline compound with Pu22 and the wild-type Pu27 sequence in solution was described [21]. The latter study revealed the small molecule bound in an “induced fit” manner at two sites on the quadruplex, with the 5’ and 3’ flanking sequences recruited to form a binding pocket for both the Pu27 and Pu22 sequences. To select the desired biologically relevant conformation, the same Pu22 sequence was further utilized in this work to obtain the reported crystal structure.

Our interest in obtaining the crystal structure of the c-MYC quadruplex formed by Pu22 stemmed from differences previously observed between solution and solid-state structures of other quadruplexes in the literature. Additionally, crystal structures often reveal other important structural information that may be absent in solution, such as the involvement of ions or water molecules important to the overall quadruplex topology, or potentially relevant quaternary interactions. On comparison of crystal and solution NMR structures of similar quadruplex sequences, differences are sometimes observed in the type of cation in the central channel and the overall topology of the quadruplexes. For example, multiple quadruplex topologies have been demonstrated in studies using the sequence repeat found in human telomeres, d(GGGTTA). Solution NMR studies have revealed that an anti-parallel quadruplex is formed in the presence of sodium ions, and alternatively, a 3+1 hybrid-type quadruplex is observed in solution containing potassium ions, with three parallel strand-edges and one anti-parallel [22–24]. In contrast, the reported crystal structure of the same sequence adopts a fully parallel arrangement with potassium cations occupying the central channel [25]. This parallel topology was also shown to be favored in studies of solutions containing molecular crowding conditions [26,27]. However, this is not always the case: for example, the c-KIT oncogene promoter quadruplex in both solution NMR and crystal structures adopts a strictly parallel form containing potassium in the central channel, with the position of the nucleotides highly-conserved [28–30]. This led us to question whether the crystal structures of other oncogene promoter quadruplexes would be analogous to their NMR solution counterparts, or if they would differ significantly as is seen with the telomeric sequences. Knowledge of the distinct topology of these quadruplexes is crucial to the design of small molecules that stabilize their structures, and there is evidence that subtle differences in structural features could allow for selectivity between specific quadruplex targets [9,31].

Here, we report a high-resolution crystal structure of the major Pu22 quadruplex formed in the human c-MYC promoter and describe several features observed that are potentially important for small molecule binding and quadruplex stabilization. We compare the crystal structure of Pu22 with previously reported solution NMR structures in an effort to inform future design of quadruplex-targeted compounds [18,32,21,33]. In addition, we have examined the features of the Pu22 crystal structure in conjunction with other quadruplex crystal structures to probe for similar features including positions of ions, water molecules and quaternary interactions. This research aims to aid future development of novel quadruplex-targeted compounds and provide information helpful in co-crystallization studies with molecules designed to bind the c-MYC promoter and other quadruplex structures.

## Materials and methods

### Crystallization

The 22-residue DNA oligonucleotide (5’-TGAGGGTGGGTAGGGTGGGTAA-3’) was synthesized and purified by Integrated DNA Technologies (standard desalting). The oligonucleotide was diluted into a stock concentration of 10 mM in DNAse/RNAse free water and the concentration was determined using a Thermo Scientific NanoDrop spectrometer. The oligonucleotide was then diluted to 2.0 mM in 20 mM sodium cacodylate buffer at pH 6.5 containing 30 mM KCl and annealed by heating for 10 minutes at 95° C and cooled overnight at 4° C prior to crystallization experiments. Initial crystal screening was done in a 96-well plate format using an Art Robbins Instruments GRYPHON liquid-handling crystallization robot. Various precipitants (PEGs, 2-Methyl-2,4-pentanediol (MPD)) were screened along with varying concentrations of salts (NaCl, KCl, LiCl etc.) as previously outlined [34]. Crystals used for diffraction data collection were grown in a 24-well plate format using the hanging-drop vapor diffusion technique with 300 mM KCl, 50 mM LiCl, and 22.5% MPD in 50 mM sodium cacodylate at pH 6.5 with a 1:1 ratio of oligonucleotide to reservoir solution. Crystals were harvested using the reservoir solution or additional MPD (30%) as a cryo-protectant and flash cooled in liquid nitrogen for storage prior to data collection.

### Data collection and refinement

Initial diffraction screening was performed using a Rigaku MicroMax 007HF X-ray generator with VariMax HighFlux optic and R-AXIS IV image plate detector. Diffraction data for structure determination were taken at the Stanford Synchrotron Radiation Lightsource (SSRL) beam line 12–2. The native dataset was collected at a wavelength of 0.9793 Å over a 360° range with 0.2° rotation per image. Data were processed using XDS with autoxds script at SSRL [35]. These data were analyzed using Xtriage in Phenix prior to phasing [36]. Initial phases were obtained by molecular replacement using Phaser in Phenix using the truncated guanine decks of a CKIT-1 promoter quadruplex as a search model (PDBID: 4WO2) [29]. An improved, complete model was constructed through iterative cycles of refinement, phasing, and manual model building using Phenix and Coot, respectively [37]. Refinement was performed in Phenix refine, followed by submission to the PDB Redo webserver [38]. The structure obtained from the PDB Redo webserver was then re-refined in Phenix refine with simulated annealing and randomized atomic displacement parameters to reduce any potential bias in *R*_free_. The final structure was refined in Phenix using data from 34.91–2.35 Å with a final *R*_work_, *R*_free_ of 0.220 and 0.245, respectively (Table 1). Atomic coordinates and structure factors are deposited in the RCSB Protein Databank with ID 6AU4 [39]. Visualization and RMSD calculations were performed using Pymol (http://www.pymol.org) [40]. Pearson correlation coefficient (CC) was calculated by randomly assigning the experimental reflection data to two half-datasets (x, y) as described by Karplus and Diederichs (Table 1) [41].

**Table 1:** Data collection and refinement statistics.

Sequence	5’-TGAGGGTGGGTAGGGTGGGTAA-3’
**Data Collection**	
Space group	P 2_1_ 2_1_ 2
Unit cell dimensions (*a*, *b*, *c*) (Å) *α*, *β*, *γ* (°)	65.7 69.8 33.0 90 90 90
Wavelength (Å)	0.9793
Resolution (Å)*	34.91–2.35 (2.43–2.35)
Total reflections*	85280 (8398)
Unique reflections*	6774 (652)
Multiplicity*	12.6 (12.9)
Completeness (%)*	99.4% (99.7%)
I/σ*	33.9 (4.4)
Wilson B-factor (Å^2^)	58.8
*R*_meas_^†^*	0.042 (0.605)
CC_1/2_^‡^*	1.000 (0.966)
**Refinement**	
Resolution (Å)*	34.91–2.35 (2.53–2.35)
*R*_work_^§^*	0.220 (0.319)
*R*_free_^§^*	0.245 (0.322)
Macromolecules	2
DNA Residues	44
Total Atoms	956
Potassium ions	6
Waters	16
Average Overall B-factor (Å^2^)	80.2
Average B-factor DNA Residues (Å^2^)	80.7
Average B-factor Potassium Ions (Å^2^)	53.1
Average B-factor Waters (Å^2^)	59.7
RMS (bonds) (Å)	0.009
RMS (angles) (°)	1.08
TLS groups	2
**PDB ID**	6AU4

*Statistics in parentheticals are for the high-resolution shell

^†^ $R_{meas}=\frac{\sum_{hkl}\sqrt{\frac{n}{n-1}}\sum_{i=1}^{n}{|I}_{i}(hkl)-\bar{I}(hkl)|}{\sum_{hkl}\sum_{i=1}^{n}I_{i}(hkl)}$, where *I*_*i*_*(hkl)* is the *i*th observation of the intensity of the reflection *hkl* and *n* is the multiplicity.

^§^ $R_{work}=\frac{\sum_{hkl}\left|{|F}_{obs}|-{|F}_{calc}|\right|}{\sum_{hkl}{|F}_{obs}|}$, where *F*_*obs*_ and *F*_*calc*_ are the observed and calculated structure-factor amplitudes for each reflection *hkl*. *R_free_* was calculated with 10% of the diffraction data that were selected randomly and excluded from refinement.

^‡^CC_1/2_ is the intra-dataset Pearson correlation coefficient (CC) calculated by randomly assigning the experimental reflection data to two half-datasets (x, y). $CC=\frac{\sum_{i=1}^{n}(x_{i}-\bar{x})-(y_{i}-\bar{y})}{\sqrt{{\sum_{i=1}^{n}\left(x_{i}-\bar{x}\right)}^{2}}\sqrt{{\sum_{i=1}^{n}\left(y_{i}-\bar{y}\right)}^{2}}}$

### Circular dichroism spectroscopy

Circular dichroism spectra were measured using a Jasco J-810 spectropolarimeter or on a Chirascan CD spectrophotometer at room temperature in a quartz cuvette with a 10 mm or 4 mm pathlength for sample A or B, respectively. The oligonucleotide sample was made at 2 μM concentration with 300 mM KCl, 50 mM LiCl, and 22.5% MPD in a 50 mM sodium cacodylate buffer at pH 6.5 to mimic the crystallization conditions (A) or at 5 μM in 10 mM potassium phosphate buffer pH 6 to replicate the conditions used in previous studies [21,42] (B). The solutions were annealed by heating to 95° C for 10 minutes and cooled overnight to 4° C prior to data collection.

## Results

**Fig 2:**
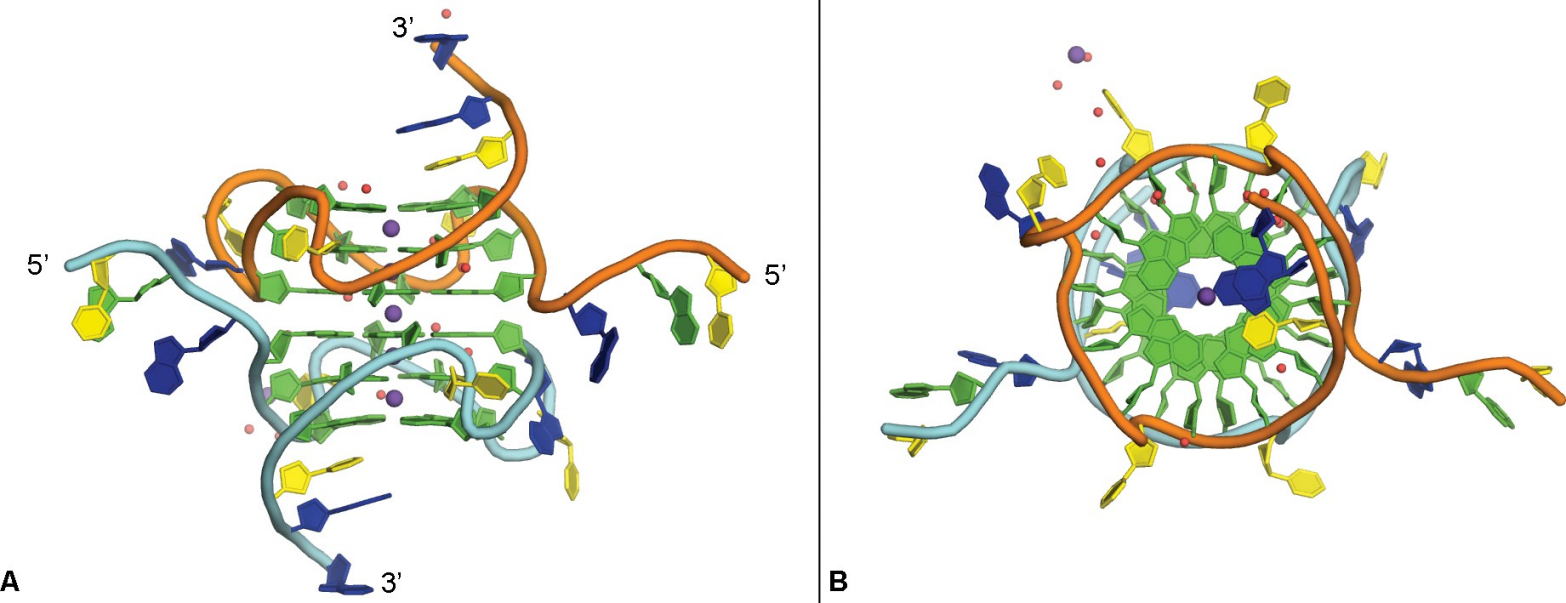
Crystal structure of c-MYC quadruplex. Side-view (A) and top-view (B) of the crystal structure of the c-MYC promoter quadruplex formed by Pu22, guanine bases in green, thymine in yellow, adenine in blue. Potassium ions in purple (two shown positioned behind strand backbones), waters in red. Orange/cyan backbone coloring represents strand A and B, respectively.

The 22 residue c-MYC promoter sequence Pu22 crystallized in the P2_1_2_1_2 space group in a stacked dimer formation with two parallel, single-stranded quadruplex structures per asymmetric unit (Table 1 and Fig 2). The two independent quaduplexes (A, B) are structurally similar with the majority of the core guanine-residue positions being maintained between the strands (G4-G6_A/B_, G8-G10_A/B_, G13-G15_A/B_, and G17-G19_A/B_ RMSD = 0.190 Å). Each individual quadruplex contains three loops and a 5’-head and 3’-tail region. The 5’-head region consists of residues T1-G2-A3 and is observed stretched away from the central G-quartets with the residues roughly orthogonal relative to the core guanines. The two related 5’-head regions appear in a quasi-mirror-related arrangement with the related atomic positions maintained (T1-G2-A3_A/B_, RMSD = 0.568 Å). The 3’-tail regions of the two quadruplexes in the asymmetric unit are comprised of residues T20-A21-A22 and are positioned below and approximately planar to the central G-quartets. The quadruplex structure also contains three double-chain reversal propeller-type loops, with two consisting of single thymine residues (T7, T16) that flank a third, two-residue loop (T11-A12). These regions display less similarity between quadruplex A and B, owing predominately to the loop formed by residues T11 and A12 (T7_A/B_, T16_A/B_, and T11-A12_A/B_ RMSD = 2.182 Å). All of the loops, the head, and the tail regions are involved in interactions important for the crystal packing. Electron density is also observed for a non-channel potassium ion and several water molecules.

### Potassium ions

**Fig 3:**
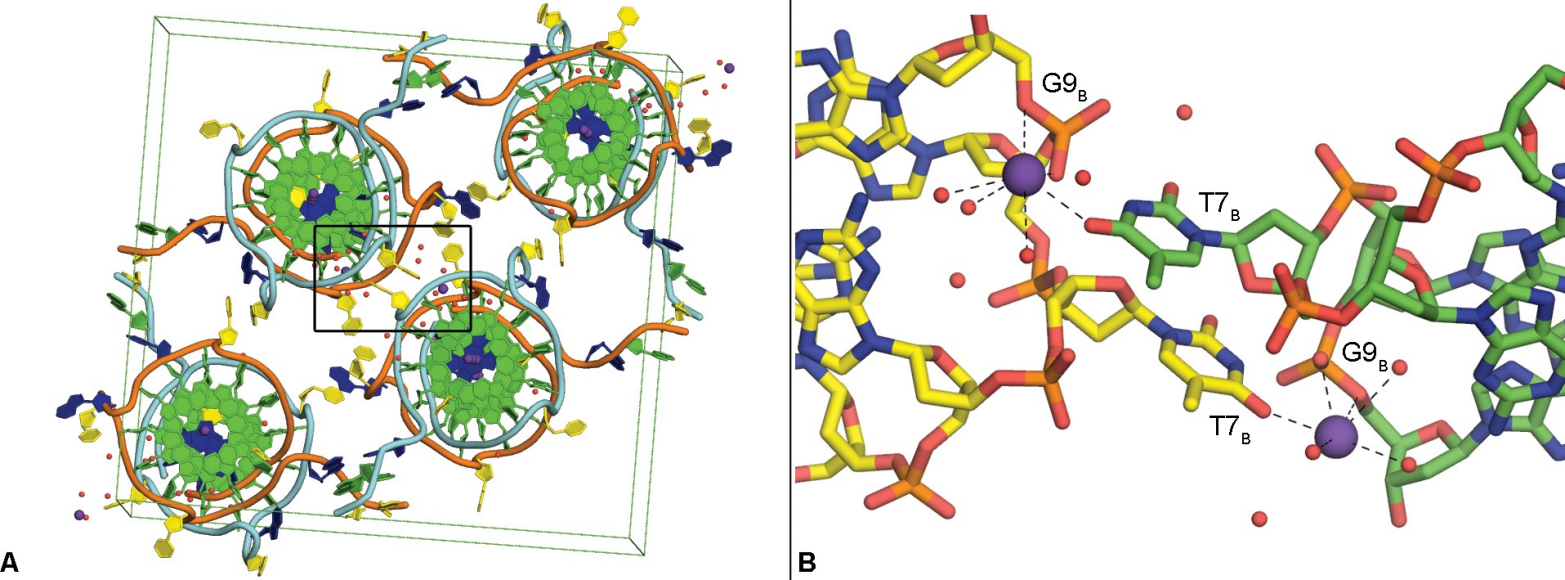
Crystal packing and non-channel potassium ions. Packing arrangement in the unit cell of c-MYC promoter quadruplex crystal structure formed by Pu22, coloring scheme same as Fig 2; black box indicates region shown in Fig 3B (A). Non-channel potassium ions interact with residues T7 and G9 (B).

Each individual quadruplex contains two potassium ions at the centers of two stacked G-quartets and an additional potassium ion similarly positioned at the interface that forms the dimer in the asymmetric unit, for a total of five potassium ions in the central cavity (Fig 2). The positions of these potassium ions relative to the G-quartets is consistent with that reported for other quadruplex crystal and NMR structures [28,32]. The ions central to the G-quartets are observed in symmetric square antiprismatic coordination with the O6 atoms of the guanine residues, as expected, with an average bond distance of 2.65 Å. An additional potassium ion is positioned between two adjacent symmetry-related strand B quadruplexes, interacting with O4 oxygen of T7 of one and backbone phosphate of G9 of the other (Fig 3B). This ion is also observed in close proximity (< 3.7 Å) to three additional water molecules. This additional potassium ion was placed following careful consideration of each of the other components present in the crystallization conditions.

### Packing interactions

**Fig 4:**
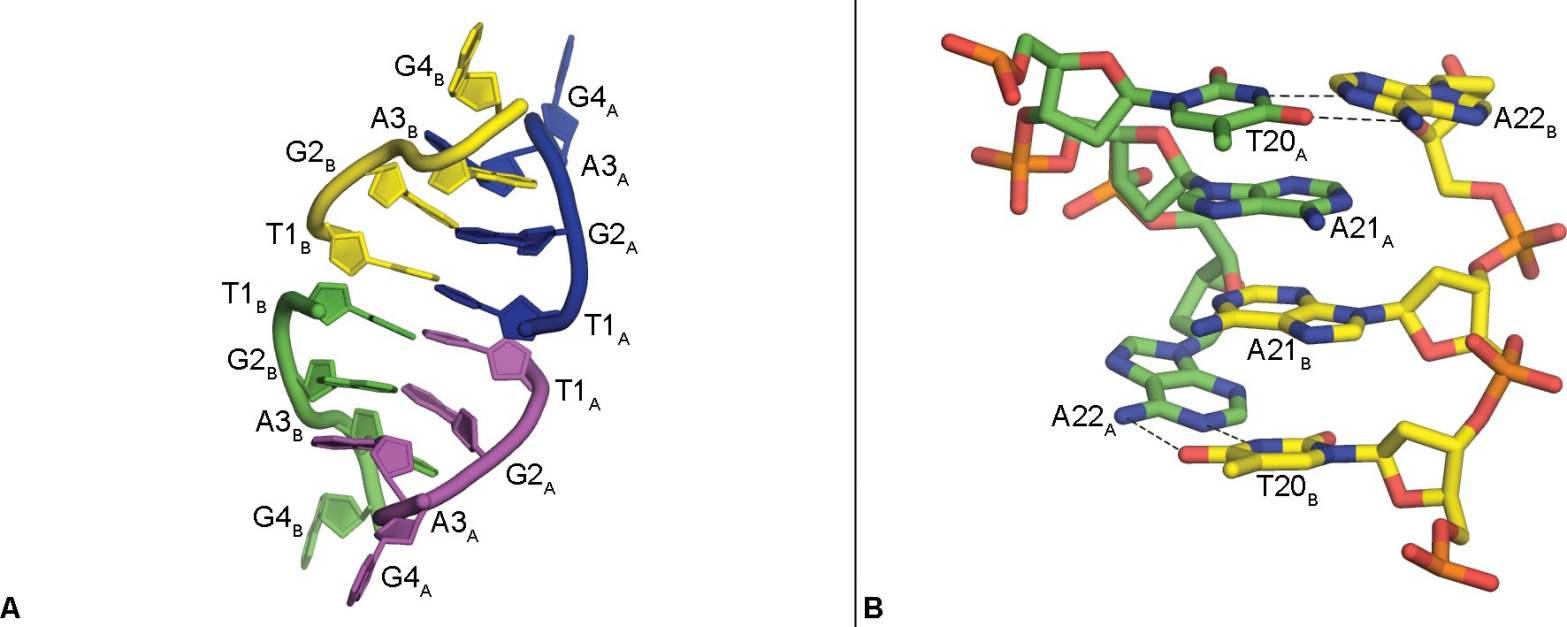
Packing interactions. Quaternary interaction of four 5’-head regions (T1-G2-A3); each color represents a separate symmetry-related strand (A). Stacked helical structure formed between 3’-tail regions of (T20-A21-A22) in the crystal lattice (B).

The Pu22 c-MYC promoter quadruplex structure displays several interesting packing interactions that induce a more elongated conformation than that observed for the same sequence by solution NMR. The two quadruplex strands, A and B, π–π stack together at the G-quartets near their 5’-ends with the G-quartets containing residues G4, G8, G13, and G17 of each quadruplex interacting to create the extended dimer structure (Fig 2). Residues T1-G2-A3 from the 5’-head of four quadruplexes in the lattice, two of A and two of B, form stacked structures stretched away from the central G-quartets, with base pairing between T1_A_/T1_B_, G2_A_/G2_B_ and G3_A_/G3_B_ (Fig 4A).

This exposes the G-quartet composed of residues G4, G7, G12, and G17, allowing the stacking of the two quadruplexes head-to-head in the asymmetric unit (Fig 2). Residues T20-A21-A22 from the 3’-tail of the two quadruplexes, A and B, interact forming a near-planar double helical structure (Fig 4B). Residue T20_A/B_ and A22_A/B_ form Watson-Crick base pairs with slightly elongated hydrogen bonds and A21_A_ and A21_B_ are involved in an apparent π-stacking interaction.

### Conserved water molecules

**Fig 5:**
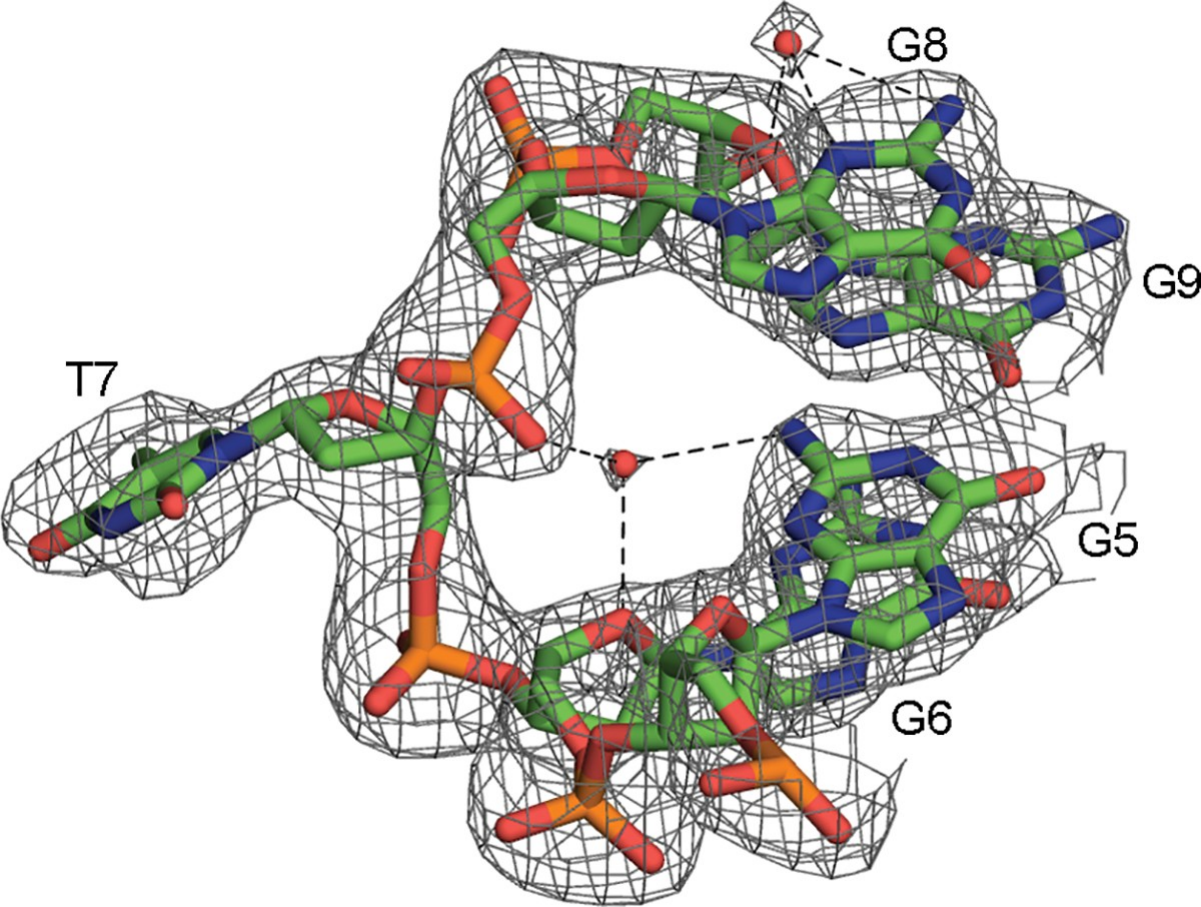
Conserved water molecules. Water molecules in conserved positions between strand A and B of the c-MYC promoter crystal structure (only strand A shown as an example), 2Fo-Fc map shown contoured at 1.0 σ.

Two water molecules were observed in nearly identical positions in each of the independent quadruplexes formed by strand A and B (Fig 5). The conserved positions of these waters suggests their presence may be important in stabilizing the quadruplex secondary structure. The first water is seen interacting with O4’ of residue G6, OP1 of G8 and N2 of G5. The second water is seen in interaction with N2/N3 of residue G8 and O4’ of G9. These waters were placed based on the presence of positive density observed in the difference map. More bound waters of this type may be present, however, the high B-factor of the data did not allow for confident placement of additional atoms even at this near-atomic resolution.

### Comparison of the crystal and solution structures of Pu22

**Fig 6:**
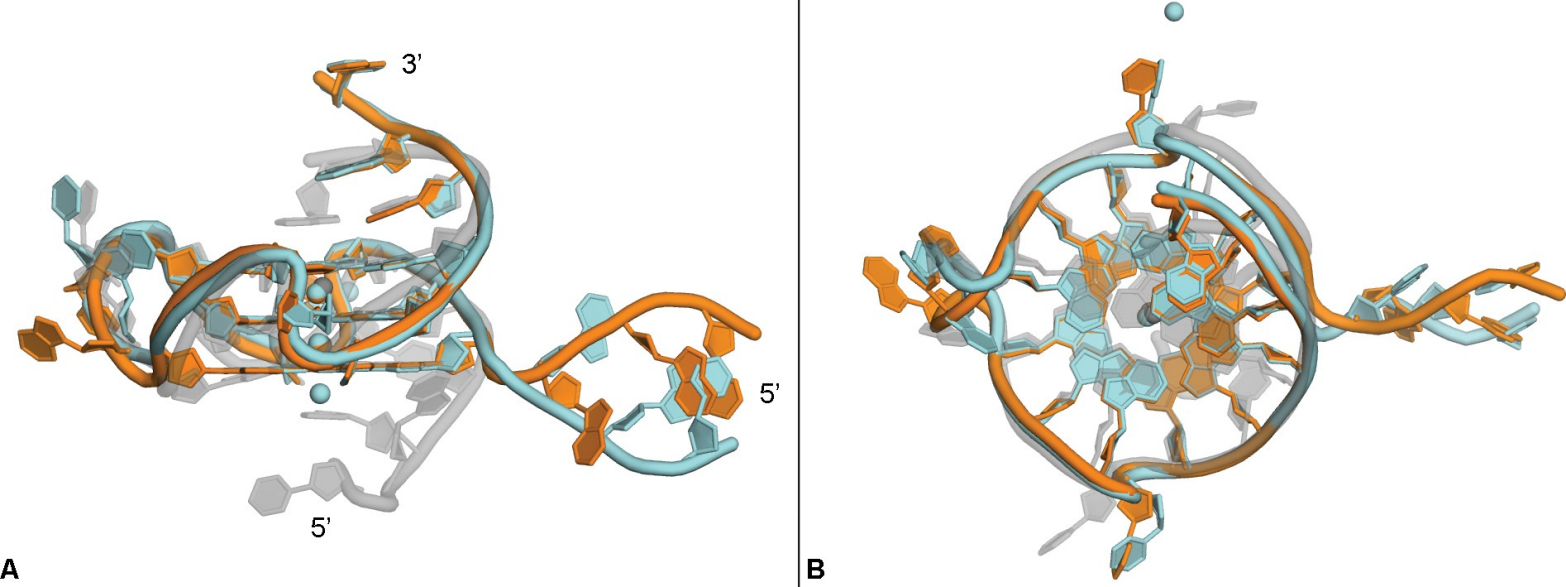
Comparison to NMR solution structure. Overlay of NMR solution structure (PDB:1XAV, grey) with strand A (orange) and strand B (cyan) of the c-MYC promoter crystal structure, side view (A), top view (B).

The overall topology of the crystal structure is consistent to that reported for the same sequence by solution NMR with several notable differences (Table 2, PDB:1XAV). The most striking difference between the solution and crystal structure is observed in the T1-G2-A3 region at the 5’-head of the quadruplexes (Fig 6). In the NMR structure, these residues lay stacked on top of the G-quartet composed of residues G4, G7, G12, and G17. ([18], PDB: 1XAV). In contrast, in the crystal structure this 5’-head region is observed extended and the top, nearest, G-quartet is the interface where the dimer is formed. The difference in this region in comparison to the NMR structure is also likely responsible for the high RMSD observed in the position of loop residue T7 and differences in the general shape of the phosphate backbone in this region. The 3’-tail region is also extended, albeit to a lesser degree, away from the G-quartet comprised of residues G6-G10-G15-G19 in the crystal structure in comparison to the NMR structure. The difference in the position of loop residue T16 can also likely be attributed to this extended conformation that is involved in crystal packing interactions.

**Table 2:** RMSD of strand A of Pu22 (PDB: 6AU4) to published DNA quadruplex structures.

RMSD (Å)			
	c-MYC (NMR, PDB: 1XAV)	c-KIT (X-ray, PDB: 4WO2)	HTelo (X-ray, PDB: 4FXM)
G-quartets*	1.36	6.65	1.03
T1-G2-A3 (5’-head)	4.16	-	-
T20-A21-A22 (3’-tail)	3.45	-	-
T7, T11-A12, T17 (loop)	4.34	-	-
Overall	7.31	10.38	2.78

*G-quartets are comprised of G4-G5-G6, G8-G9-G10, G13-G14-G15, and G17-G18-G19.

RMSD comparisons were not made between distinct quadruplex structures (c-KIT, telomeric) for the loop, head and tail regions due to significant sequence differences.

**Fig 7:**
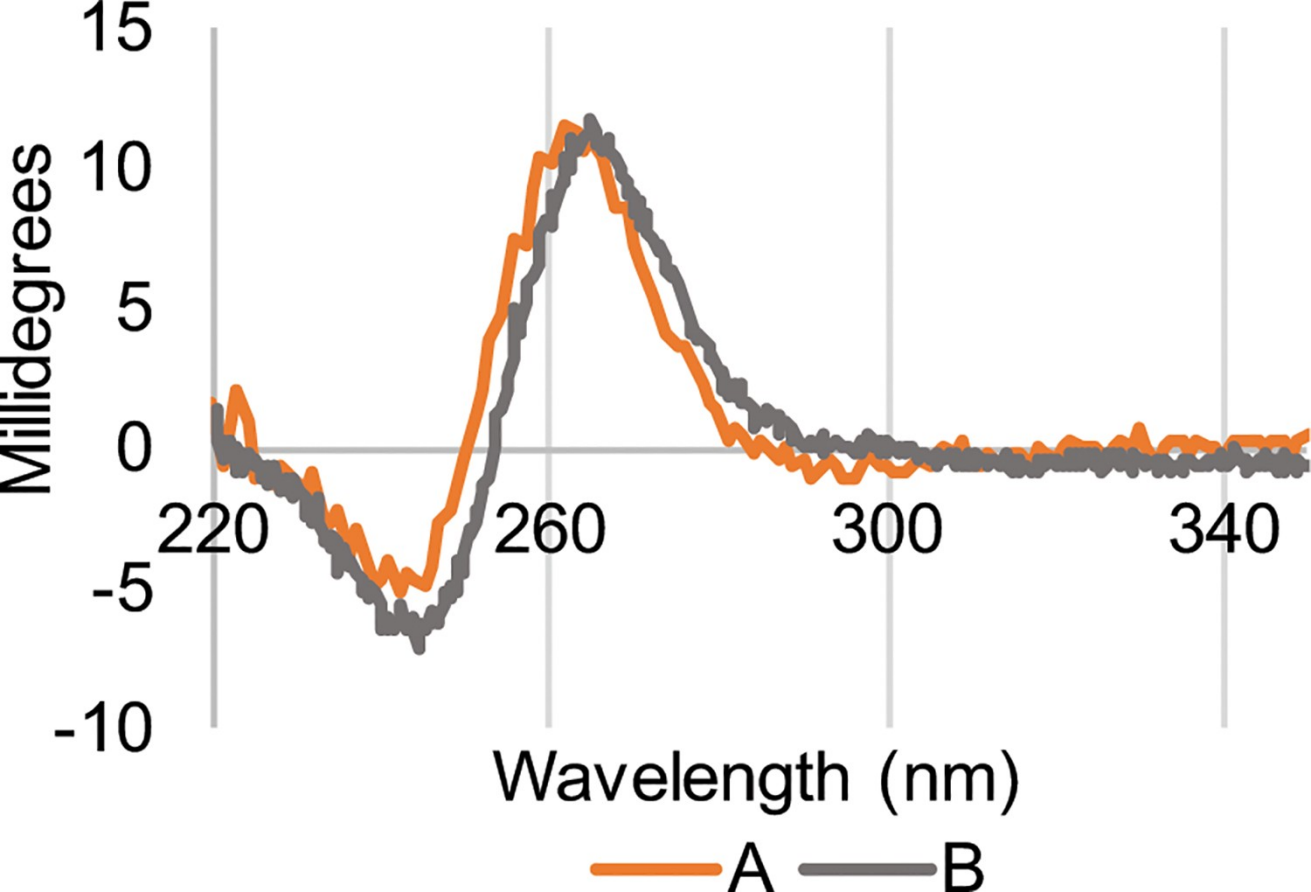
Circular dichroism spectroscopy. CD spectra of the Pu22 oligonucleotide under crystallization conditions (A) or conditions previously reported to form the parallel c-MYC promoter quadruplex (B).

To examine the effect of the crystallization conditions on the conformation of Pu22, we performed circular dichroism spectroscopy at conditions analogous to those used in solution NMR studies [21]. Parallel quadruplex structures display characteristic peaks at approximately positive 265 nm and negative 240 nm in their CD spectra [1]. The parallel topology was confirmed for the Pu22 oligonucleotide under both the conditions used in the solution NMR studies as well as at the crystallization conditions to provide additional evidence the conformation observed in the crystal structure is not an artifact of the crystallization environment (Fig 7).

### Comparison to other crystal structures

**Fig 8:**
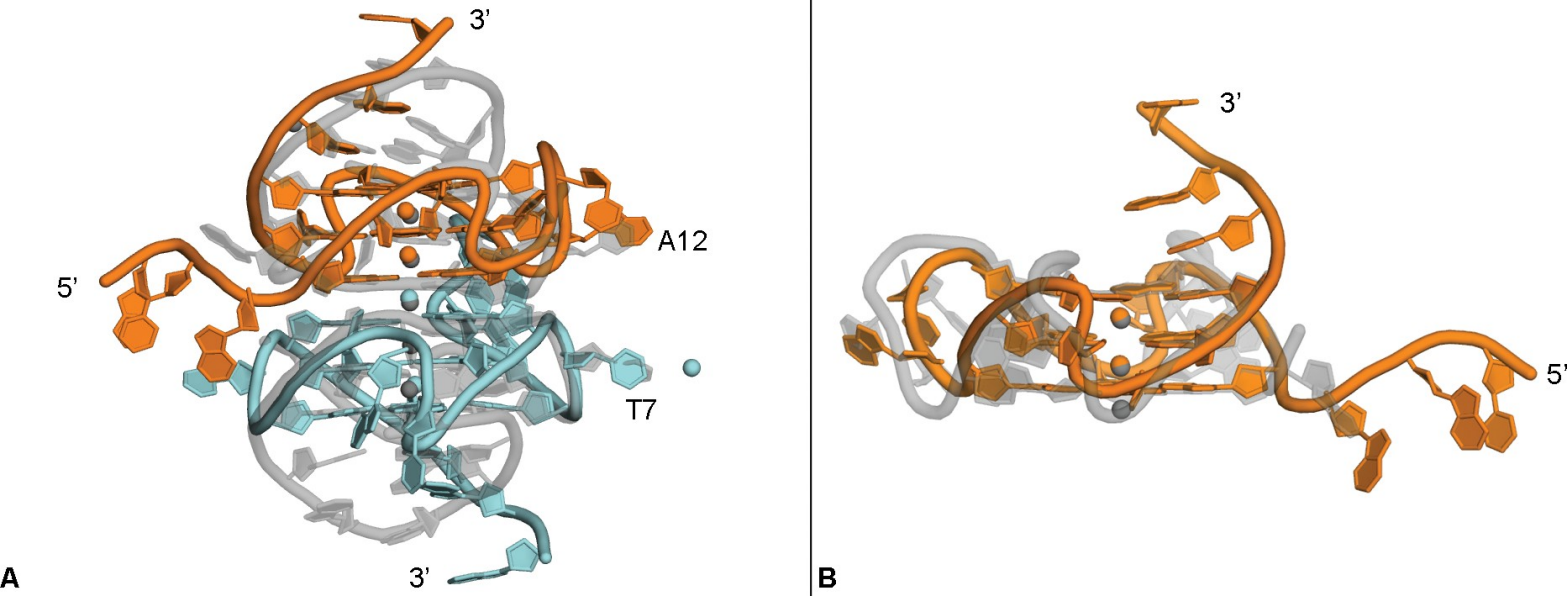
Comparison to other quadruplex crystal structures. Overlay of c-KIT crystal structure (PDB:4WO2, grey) with strand A (orange) and strand B (cyan) of the c-MYC promoter crystal structure; loop residue labels correspond to c-MYC crystal structure (A). Overlay of human telomeric crystal structure (PDB: 4FXM, grey, ligand not shown) with strand A (orange) of the c-MYC promoter crystal structure (B).

The human c-KIT promoter region also contains a quadruplex sequence motif, and its crystal structure has some distinct similarities and differences to the c-MYC quadruplex formed by Pu22 (Fig 8A and Table 2, PDB: 4WO2). In both crystal structures, a quadruplex dimer forms the asymmetric unit, with the G-quartets closest to the 5’-region observed in a stacked formation. The c-KIT strands (A & B) forming the dimer are rotated one guanine relative to one another (approximately 90°), whereas the c-MYC strands (A & B) are rotated two guanines relative to one other (approximately 180°). This causes the c-KIT quadruplex loop region nucleotide C9_A_ to occupy the same space as loop nucleotide A12_A_ of the c-MYC quadruplex, and this is regardless of the additional T11_A_ present in this loop region of the c-MYC structure. The loop nucleotide C9_B_ of c-KIT superimposes with loop nucleotide T7_B_ of the c-MYC quadruplex. The c-MYC quadruplex structure contains a non-channel potassium ion interacting with residues T7 and G9. A non-channel potassium ion is also observed at an unrelated site in the c-KIT crystal structure interacting with residues A16 and G17.

Several crystal structures of the fully parallel quadruplex formed by the human telomeric repeat sequence in complex with a small molecule ligand have been reported, including an example of the intramolecular arrangement at 1.65 Å (Fig 8B, PDB: 4FXM) [43]. The telomeric quadruplex forms a dimer with both strands reported as identical in the asymmetric unit. The telomeric structure is remarkably similar to the Pu22 c-MYC quadruplex with the exception of the loop regions. In the c-MYC structure, these loops are formed by one or two nucleotides, whereas the telomeric structure contains loops made of three nucleotides each. However, even with this difference in loop region length, the c-MYC quadruplex can be superimposed on the telomeric quadruplex with an RMSD of only 2.78 Å (Table 2, PDB: 4FXM). Interestingly, the Watson-Crick packing interaction seen with the T20-A21-A22 tail of two c-MYC quadruplexes is also observed in the crystal structure of the telomeric quadruplex, but between two loop regions containing residues T11-A13 and A1, suggesting that a 3’-terminal TAA sequence may be useful for crystallization of other quadruplexes [43].

During preparation of this manuscript, a 3.8 Å structure was reported of a sequence modified c-MYC promoter quadruplex in complex with the DEAH/RNA helicase DHX36 [44]. In the reported complex structure, it is suggested quadruplex destabilization by the helicase results in a one-residue shift of the nucleotides involved in formation of the three G-quartets, causing the quartet nearest to the 5’-head region to be reformed by G4, G8, A12, and T16. This finding is noteworthy, however, due to the low-resolution of the structure and rearrangement observed in the presence of the helicase, it did not appear relevant for comparison with our crystal structure.

## Discussion

In this manuscript we report a high-resolution crystal structure of the major quadruplex formed in the promoter region of the human c-MYC oncogene. The overall topology is in general agreement with the previously published solution NMR structures, with the major differences occurring predominately in the head and tail regions of the sequence and not in the central G-quartet structure [18,21]. This is important as it strengthens the validity of previous and ongoing computational and synthetic studies that have used the NMR solution structures as a guide.

The more exposed G-quartets observed in the crystal structure, in comparison to those reported using solution NMR, confirm both the flexibility of the head and tail regions of the promoter sequence and the rigid nature of the G-quartets in this quadruplex. This could be biologically relevant as the flanking regions would not be terminal residues in genomic DNA and are likely able to project away from the G-quartets as is observed in the crystal structure (Fig 2). This will be important in future studies with the goal of developing small molecule ligands to bind the c-MYC quadruplex. For example, in the solution NMR study of a small molecule interaction with the c-MYC promoter quadruplex, it was observed that part of the ligand binding pocket involved nucleotides in the 5’-head (A3) and 3’-tail regions (T20, A21) (PDB: 2L7V, [21]). It is possible, as suggested by the c-MYC quadruplex crystal structure, that these residues are not in close proximity to provide such interactions in the biological setting as illustrated schematically in Fig 1. In this scenario, the reported “induced fit” mode of ligand binding would require major structural rearrangement and be less energetically favorable. Therefore, an alternative strategy would be to design ligands with a binding mode that takes advantage of the more linearized conformation of the head and tail regions, potentially affording tighter binding and selectivity for the c-MYC quadruplex. This alternative binding hypothesis will be a crucial consideration in the future design of c-MYC quadruplex-targeted small molecule ligands.

The packing interactions observed in the crystal highlight the importance of the loop, head and tail regions of the sequence to allow for crystal formation and will be generally helpful in optimizing quadruplex sequences for crystallization. The Watson-Crick bonding observed at the 3’-tail of the Pu22 sequence also suggests a preference for nucleotides not involved in the G-quartet core region to follow the normal B-DNA base pairing paradigm even in very close proximity to the G-quartets. However, there is previous evidence that these flanking regions may remain single-stranded under conditions of negative supercoiling [17].

Comparison of the c-MYC quadruplex crystal structure to the c-KIT quadruplex and the human telomeric quadruplex show the majority of differences occur in the head, tail and loop regions. Differences in the length of loop regions and position of the loop nucleotides will provide useful tools for designing ligands that would be selective for specific quadruplex structures as has been suggested previously [9,45]. Overall, this work has provided the basis for ongoing co-crystallization studies with the Pu22 c-MYC promoter quadruplex and our set of novel quadruplex ligands, the anthracenyl isoxazole amides (AIMs) [33,46]. These findings will also be of benefit to other researchers in their efforts to target the c-MYC promoter and other therapeutically relevant quadruplex targets.

## Supporting information

S1 Fig G-quartet potassium ions. Example of square antiprismatic coordinated potassium ions central to two stacked G-quartets in the crystal structure.
(TIF)

S1 File 6AU4 PDB validation report. (PDF)

S2 File 6AU4 molecular coordinates. (PDB)

S3 File 6AU4 x-ray data. (MTZ)
