# Supplementary figures and images for: Crystal structure of the major quadruplex formed in the promoter region of the human c-MYC oncogene
Source: PLoS One. 2018 Oct 12;13(10):e0205584. doi: 10.1371/journal.pone.0205584 (PMC6185859; doi:10.1371/journal.pone.0205584)

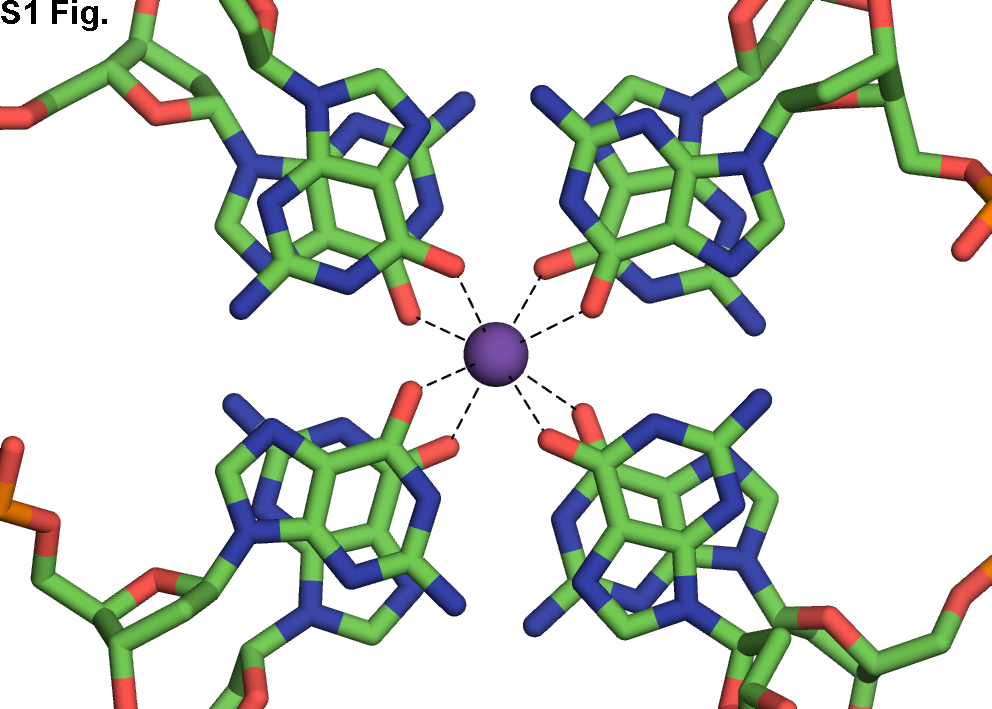

Supplement: S1 Fig — Example of square antiprismatic coordinated potassium ions central to two stacked G-quartets in the crystal structure. (TIF) [file pone.0205584.s001.tif]
